# Interoceptive–reflective regions differentiate alexithymia traits in depersonalization disorder

**DOI:** 10.1016/j.pscychresns.2013.05.006

**Published:** 2013-10-30

**Authors:** Erwin Lemche, Michael J. Brammer, Anthony S. David, Simon A. Surguladze, Mary L. Phillips, Mauricio Sierra, Steven C.R. Williams, Vincent P. Giampietro

**Affiliations:** a Section of Cognitive Neuropsychiatry, Institute of Psychiatry, De Crespigny Park, London SE5 8AF, UK; b Department of Neuroimaging, Institute of Psychiatry, De Crespigny Park, London SE5 8AF, UK; c Western Psychiatric Institute and Clinic, University of Pittsburgh, 3811 O'Hara Street, Pittsburgh, PA 15213, USA

**Keywords:** Depersonalization Disorder, Functional magnetic resonance imaging, Facial expressions, Differential regression analysis, Toronto Alexithymia Scale, Functional connectivity

## Abstract

It is unclear to what degree depersonalization disorder (DPD) and alexithymia share abnormal brain mechanisms of emotional dysregulation. We compared cerebral processing of facial expressions of emotion in individuals with DPD to normal controls (NC). We presented happy and sad emotion expressions in increasing intensities from neutral (0%) through mild (50%) to intense (100%) to DPD and non-referred NC subjects in an implicit event-related fMRI design, and correlated respective brain activations with responses on the 20-item Toronto Alexithymia Scale (TAS-20) and its three subscales F1-F3. The TAS-20 predicts clinical diagnosis of DPD with a unique variance proportion of 38%. Differential regression analysis was utilized to ascertain brain regions for each alexithymia subscale. Differential regions of total alexithymia severity for happy emotion were the globus pallidus externus; for identifying feelings (TAS-20 F1 subscale), the right anterior insula; for description of feelings (F2), the right dorsal mid-anterior cingulate gyrus (BA 24); and for externally oriented cognitive style (F3), the left paracingulate gyrus (BA 32). For sad emotion, the differential region for the total TAS-20 score was the dorsal anterior cingulate gyrus (BA 24); for TAS-20 F1, the left inferior anterior insula; for TAS-20 F2, the right PCC (BA 31); and for TAS-20 F3, the right orbital gyrus (BA 10). Supporting our hypotheses, the ascertained brain regions for TAS-20 subscales subserve interoception, monitoring and reflection of internal states and emotion. The presented analyses provide evidence that alexithymia plays a substantial role in emotional dysregulation in DPD, presumably based on restrictions in interoception.

## 1 Introduction

Alexithymia is a cognitive trait, which has been implicated in emotion dysregulation in a number of mental, somatoform and somatic health problems. Alexithymia is associated with reduced introspective awareness and a lack of emotional reasoning towards others and the self (Taylor, 1984). As a consequence, verbal expression of emotions and cognitive reflection of emotional processes is impaired, and this leads to a tendency to respond with unmoderated physiological arousal states towards external events (Spitzer et al., 2005). The higher autonomic reactivity is generally seen as an adverse disposition that profoundly contributes to stress-related mental and somatic disorders. A disposition towards heightened internal arousal due to increased physiological reactivity has also been demonstrated to accompany more extreme cognitive and moral tendencies (Oxley et al., 2008).

The alexithymia syndrome was introduced as a research construct by the psychiatrist Sifneos in 1972 (Taylor, 1984), and later operationalized by Taylor and colleagues as a self-report instrument, the Toronto Alexithymia Scale (TAS-26 and TAS-20, with its factor-analytic subscales F1-F3), which is at present the most widely accepted research measure. The TAS-20 subscale F1 reflects the ability to internally discriminate and identify feelings and emotions. The TAS-20 subscale F2 consists of self-report items quantifying the capability to cognitively represent and to verbalize feelings and emotions. Finally, the F3 subscale of the TAS-20 contains self-descriptions endorsing the inclination to maintain an outward direction of attention with respect to material objects.

Recent longitudinal developmental research has also yielded evidence to support the notion that the alexithymia trait may consist of a neurodevelopmental cognitive deficit (Lemche et al., 2004), which might explain its lifetime endurance. Moreover, overlaps have recently been highlighted to exist between alexithymia and autism spectrum disorders, e.g. in the lack of emotion-related cognition, incapability of introspection, and deficits in processing reflections on significant others (Fitzgerald and Molyneux, 2004). In support of this conjecture, a study in autism spectrum disorder found that empathy deficits observed in autism may be due to the large co-morbidity between alexithymic traits and autism (Bird et al., 2010). As has been argued, both developmental disorders share common impairments in self- and other-related mentalization. Furthermore, a view according to which alexithymia could be a neurologically driven process has recently received strong support by the replicated neuropsychological finding that alexithymia appears regularly as a consequence of traumatic brain injury (Wood and Williams, 2007, Wood et al., 2009). As is well documented, alexithymia is typically found concomitant to somatization states, depression, anxiety, cardiovascular problems and affective disorders in large population samples (Wood et al., 2009).

Emotional dysregulation is also a key feature in DPD, a syndrome often subsumed under dissociation (Sierra, 2010). Typical co-morbidities of DPD include anxiety and depression (Mula et al., 2007, Baune et al., 2010), whereas dissociative memory impairments are not regularly found when depersonalization/derealization diagnoses are present (Baker et al., 2003). Most recent studies on DPD have revealed altered social competence based on fewer self-reported cognitive empathic abilities (Lawrence et al., 2007). The empathy deficits were found to include social anxieties alongside an increased self-orientation bias, which is reminiscent of findings in alexithymia. There are bidirectional findings in DPD regarding possible emotional memory impairments. On the one hand, DPD patients showed elevated recognition for emotive words (Montagne et al., 2007), yet lacked the usual enhancement effect for emotional memories (Medford et al., 2006). This behavioral deficit corresponds to a lack of activation in the relevant cerebral regions (Medford et al., 2006). According to previous functional magnetic resonance imaging (fMRI) studies (Phillips et al., 2001), DPD patients tend to respond with increased right ventrolateral prefrontal cortex engagement towards visual emotional stimuli, instead of activating limbic regions. Recently, alexithymia level has been deemed a strong predictor of a clinical DPD diagnosis (Simeon et al., 2009). We therefore decided to investigate in greater detail the relationship between alexithymia and DPD using an experimental fMRI study. With respect to clinical alexithymia, recent findings typically suggest a strong interrelation with dissociation with regard to posttraumatic stress, emotional numbing, and alexithymia (Frewen et al., 2008). In individuals with posttraumatic stress disorder (PTSD), TAS-20 scores correlated positively with neural responses in insula, posterior cingulate cortex (PCC), and thalamus, and negatively with response in anterior cingulate cortex (ACC) (Frewen et al., 2006).

To be able to compare cerebral mechanisms of alexithymia in DPD with normal emotional functioning, we used an emotional facial expression paradigm with fast implicit visual stimulation that resembles natural social encounters. We planned to compute correlation images to ascertain brain regions associated with the TAS-20 and its subscales. Differential regression was used to identify brain regions that differ in association with questionnaire scores, which would enable us to compare both groups with respect to differential substrates of alexithymia traits. We expected to be able to find alexithymia correlations in the above regions. In particular, for normal controls, we hypothesized correlations in the (i) paracingulate/anterior and posterior cingulate regions previously (ii) implicated in healthy controls (Berthoz et al., 2002). For DPD patients, we expected (iii) correlations in the insula, (iv) the thalamus, (v) and further regions in the pain matrix, as reported for alexithymia in clinical populations (Kano et al., 2007). For the subscales F1 and F2, specifically, we expected to find regions with interoceptive accuracy discriminating the two groups, following respective findings in normal individuals (Critchley et al., 2004): (vi) anterior insula, thalamus, operculum, and anterior cingulate. For simple correlations of subscales F1 and F2, however, we anticipated for DPD patients regions not associated with emotional awareness (vii).

## 2 Experimental procedures

### 2.1 Participants

The Joint South London Maudsley and Institute of Psychiatry Research Ethics Committee endorsed all experimental procedures. The study was conducted in compliance with the Helsinki Declaration (World Medical Association, 1991), and normal controls (NCs) were compensated for their participation. Informed consent was signed by all subjects to the scientific use of their data. Investigated was a total sample of 21 volunteers. The nine primary-diagnosis DPD patients (mean age, 36.11±7.31 SD years; education level, 2.22±0.68; 2=junior college level) consisted of five males and four females. These patients were in treatment for DPD at the Maudsley Hospital, London, in a specialized clinic (ASD and MLP). A psychiatrist not involved in the study had independently diagnosed DPD according to DSM-IV-TR (300.6) criteria, and the clinical cut-off level of >70 on the Cambridge Depersonalization Scale (CDS) discriminative for DPD (Sierra and Berrios, 2000) was exceeded for all patients (mean score 175.77±110.85). Patients were unmedicated in majority, but three of them were medicated, each with different substances (paroxetine, fluoxetine, olanzapine). Minor co-morbid dysthymic (DSM-IV-TR 300.4) and/or mild inspecific anxiety symptoms (DSM-IV-TR 300.02) were diagnosed in six patients. The DPD patients were compared to 12 normal control (NC) subjects chosen to match education, socioeconomic status, gender ratio, and general intellectual and social functioning (mean age, 27.25±4.95 years; education level, 2.58±0.79; 7 males and 5 females). All participants were right-handers according to scores on the Edinburgh Handedness Inventory (Oldfield, 1971).

### 2.2 Self-report questionnaire data

Clinical self-report forms were completed prior to MRI scans. All participants completed the Toronto Alexithymia Scale, 20-item version (Taylor et al., 1988, Taylor et al., 1990, further to the CDS clinical cutoff measure for DPD (see above). The TAS factors are replicable across cultures, with well-established psychometric properties, and are a widely accepted measure of the alexithymia construct. As described above, the three subscales of the TAS, F1–F3, quantify identification feelings and emotions (F1), description of feelings and emotions (F2), and orientation to external objects (F3). A sample statement for F1 items is: “When excited, I don't know if I am sad, anxious or angry”. An example of F2 statements is: “Others ask me to better explain my feelings”. A representative statement in F3 is: “I like to share my opinion on things”.

### 2.3 Implicit facial expression neuroimaging tasks

Subjects were presented with 20 facial expression stimuli at 0% (neutral)–50% (mild)–100% (intense) intensities of happy and sad emotion expressions. Separate scans were performed for happy and sad conditions. Subjects were required to determine the sex of the face in the implicit emotion recognition task. The exact paradigm is described in Appendix A linked to this article. The percentage of correct responses had been found to be the most accurate index in group comparisons with regard to type I error rates, statistical power and sensitivity for our ranges of experimental trial numbers (Rotello et al., 2008).

### 2.4 fMR image acquisition and analysis

Gradient echo echoplanar imaging (EPI) data were acquired on a Neurovascular GE Signa 1.5 T system (General Electric, Milwaukee, WI, USA.), equipped with 40 m/mT high-speed gradients at the Maudsley Hospital, London, UK. A quadrature birdcage headcoil was used for RF transmission and reception. 180 T_2_⁎-weighted images were acquired over 6 min for each of the two tasks at each of 16 near-axial noncontiguous 7-mm-thick planes parallel to the intercommissural (anterior commissure-posterior commissure, AC-PC) line: TE 40 ms, TR 2000 ms, in-plane resolution 3.44 mm, interslice gap 0.7 mm, flip angle (FA) α 70°, matrix 64^2^, field of view (FOV) 25 cm providing whole brain coverage. During the same session, a high-resolution EPI dataset was acquired with a gradient echo EPI pulse sequence. The structural images were acquired at 43 near-axial 3-mm-thick planes parallel to the AC-PC line: TE 73 ms, TI 180 ms, TR 16,000 ms, in-plane resolution 1.72 mm, inter-slice gap 0.3 mm, matrix size 128^2^, FOV 25 cm, FA α 90°. The high resolution EPI dataset was later used to register the fMRI datasets acquired from each individual in standard stereotaxic space. The statistical software program package *XBAM* (www.brainmap.it) developed at the Institute of Psychiatry, was used to perform the analysis of the fMRI data. XBAM combines nonparametric permutation based resampling methods with GLM statistics, wavelet signal denoising methods, control of false-positive voxels and clusters, and reports exact significances rather than results corrected for family-wise error rates. A detailed description of the fMRI analysis methods can be found in Appendix A linked to this article.

### 2.5 Correlation images

Under the assumption that the subtraction map reflects pure emotion-induced cerebral activation at higher intensity levels, 100–50% aggregate images were computed, after removing neutral facial expression activation. The aggregate images were used as a basis for correlation analysis with TAS-20 scales. The method herein used was first to compute the Pearson product-moment correlation coefficient *r* between the measured TAS-20 factor scales and blood oxygen level-dependent (BOLD) effect data, and then to compute the null distribution of correlation coefficients by permuting the BOLD data at each voxel a minimum of 50 times, and combining the data over all voxels. Age and sex were entered into all correlations as covariates. Threshold cluster level maps, where *r* is significant, could then be computed at the expected level of type I error clusters as previously described. Comparative peak signal levels (for the two groups) of BOLD signal changes in these regions were determined by extracting the effect sizes of the peak active voxels for each of the two groups.

### 2.6 Images of differences in regression slopes

The aggregate activation maps and correlation images served as a basis for the ascertainment of regions in which the two groups significantly differed for an alexithymia factor. Differential linear regression models were set up by inclusion of age and sex as covariates of no interest, and used 50 permutations per voxel to test the regression slope difference between the two groups. The α-level for the 3D cluster difference of the regression slopes was set at *p*<0.05 voxel-wise followed by a cluster level of 0.01. For individual measures of clinical traits, the extent of the difference in regression of the behavioral data and individual fMRI contrasts between two groups were computed and tested for significance. Group differences in regression slopes can be calculated at each voxel by first computing, for each group independently, the regression coefficient between the behavioral clinical data for each subject and the BOLD signal, and then by subtracting the resulting two values. To determine the significance of these differences in linear regression slopes, the appropriate null distribution was generated by randomly permuting subjects between the groups (without replacement), therefore removing group differences. For each of the permutations, the difference in regression slopes between the permuted groups was calculated and the resulting values were combined over all voxels to produce a whole brain null distribution of differences in regression.

The critical value for significance at any particular *p* value was then obtained from this distribution after simply sorting it and selecting the appropriate point from the sorted distribution. For example, the critical value for a one-tailed test at *p*=0.05 would be the value of the difference in regression coefficients in the null distribution chosen such that 95% of all the null values lay below that point. Testing can then be extended to cluster level as described previously. The cluster probability under the null hypothesis can be chosen to set the level of expected type I error clusters at an acceptable level (e.g. <1 3D cluster per whole brain). Signal levels for the differential regions so ascertained used as masks were then extracted at 6 s post-stimulus (the peak hemodynamic response time) using the aggregate activation, by choosing the most activated voxel. The extraction was done for descriptive purposes to inform about signal levels, without re-analysis of the data.

## 3 Results

### 3.1 Performance accuracy during the gender decision task

Percentage correct responses (±SD) in determination of the sex of the faces were: 0% happy, NC 46.25±12.08, DPD 48.78±11.59; 50% happy, NC 60.00±12.25, DPD 48.89±16.54; 100% happy, NC 47.50±8.66, DPD 48.33±7.91; 0% sad, NC 42.08±6.20, DPD 46.67±8.66; 50% sad, NC 55.42±10.32, DPD 58.89±11.67; 100% sad, NC 57.08±10.97, DPD 51.67±9.01. Testing for significance, judgment accuracies in the gender decision task for facial expressions yielded no significant between-group contrasts. Overall figures were in line with other studies utilizing implicit facial paradigms (Surguladze et al., 2005). No systematic differences between DPD and NC emerged for reaction times.

### 3.2 Self-report questionnaires

**Fig. 1:**
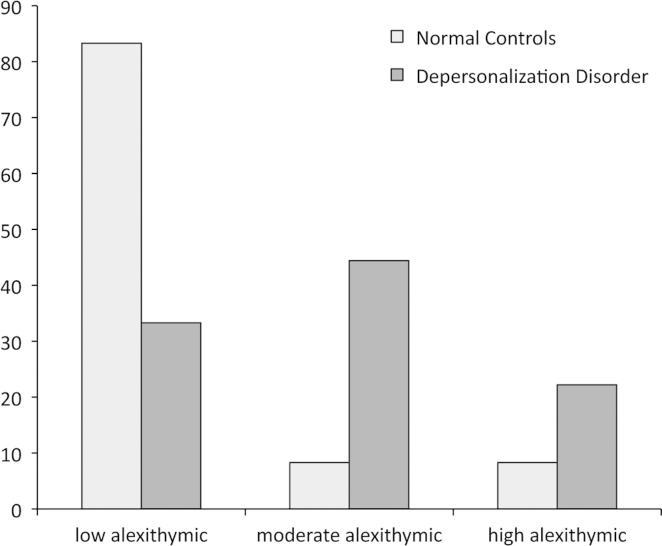
Distribution of alexithymia severity.

The descriptive values for the questionnaire data are listed in Table 1. The internal consistencies of the CDS (Cronbach's *α=*0.95) and the TAS (TAS-20 *α=*0.86, F1 *α=*0.91, F2 *α=*0.97, F3 *α=*0.87) scales were satisfactory for subsequent analyses. Significant between-group differences were observed for the Toronto Alexithymia Scale with its dimensions for Identification of Feelings (TAS-20 F1) and Description of Feelings (TAS-20 F2), and its dimension for External-Concrete cognitive style (TAS-20 F3). In all of these dimensions, scores were higher for DPD patients than for NC. These significant group differences, however, do not imply clinical cutoff levels. Alexithymia severity is currently classified as low (TAS-20 score ≤ 51), moderate (51< TAS-20 score <61), or high alexithymia levels (TAS-20 score ≥ 61) (Spitzer et al., 2005). According to this classification, the distributions pertaining to the alexithymia level in NC were 83% low, 8% moderate, and 8% high. In DPD, alexithymia levels were low 33%, moderate 44%, and high 22%. (Fig. 1). Also according these distributions, the differences in alexithymia severity between NC and DPD were significant (Wald–Wolfowitz *Z*=−3.105, exact one-tailed *p*<0.001). In sum, our NC group is strongly low alexithymic, whereas the alexithymia severity in the DPD group is by two-thirds on mid-to-high alexithymia levels, with strong clinical alexithymia severity in the upper quartile range.

**Table 1:** Comparisons of control vs. depersonalization patient groups—alexithymia self-report data.

Alexithymia and composites	Control		Depersonalization		Median test	*p*-Value
	*M*	SD	*M*	SD		
TAS-20 Mean Score	36.66	12.48	52.44	12.62	−2.85	0.010
TAS-20 Factor 1	1.603	0.43	4.460	1.12	−1.91	0.071
TAS-20 Factor 2	2.431	0.97	4.028	2.89	−1.79	0.089
TAS-20 Factor 3	2.132	0.53	4.917	6.42	−1.51	ns

*Note*: d.f.=19.

### 3.3 Interrelations of the self-report questionnaires

Age was significantly correlated at *p*<0.05 (two-tailed) with total alexithymia severity (*r*=0.46), but not with subscale scores, and with total CDS score (*r*=0.52). Gender was not significantly associated with any self-report. CDS correlated with TAS-20 (*r*=0.55), with F1 (*r*=0.54), and only marginally with F2 (*r*=0.38), but not with F3.

### 3.4 Prediction of clinical diagnoses

Given the behavioral results, we attempted to replicate the prediction of DSM diagnoses of DPD by using clinical scales, as described in the literature (Sierra and Berrios, 2000, Simeon et al., 2009). Logistic regression was used to model the linear relationships between CDS and TAS-20 and clinical diagnoses. Receiver operating characteristics (ROC) were utilized to assess the classification sensitivity of the self-report scales with respect to psychiatric diagnosis. The CDS score significantly predicted the clinical DPD diagnosis (*χ*^2^=17.453, −2 log likelihood=11.229, Wald=5.509, Nagelkerke *R*^*2*^=0.758, *p*<0.0001) (95% CIs 1.040–1.517). With CDS as classifier, the area under the ROC curve=0.97, asymptotic *p*<0.0001 (95% CIs 0.909–1.063). In addition, also the total TAS-20 score predicted significantly DPD diagnosis (*χ*^2^=7.076, −2 log likelihood=21.606, Wald=4.872, Nagelkerke *R*^*2*^=0.384, *p*<0.008) (95% CIs 1.011–1.207). However, none of the TAS-20 subscales alone or combined achieved a significant contribution to any regression equation. With TAS-20 as discrimination variable and DPD diagnosis as classifier, the area under the ROC curve=0.815, asymptotic *p*<0.016 (95% CIs 0.621–1.009). Therefore, both ROC analyses successfully refuted the null hypothesis of 0.5 in areal classification, and demonstrated sufficient specificity for the clinical diagnosis. In summary, it can be stated that although TAS-20 score is also a significant predictor of the clinical DPD diagnosis, the CDS retains a much higher unique variance explanation expressed as *R*^*2*^ in the regression equation for the depersonalization diagnosis.

### 3.5 fMRI correlation images with TAS and difference regions for regression slopes

**Fig. 2:**
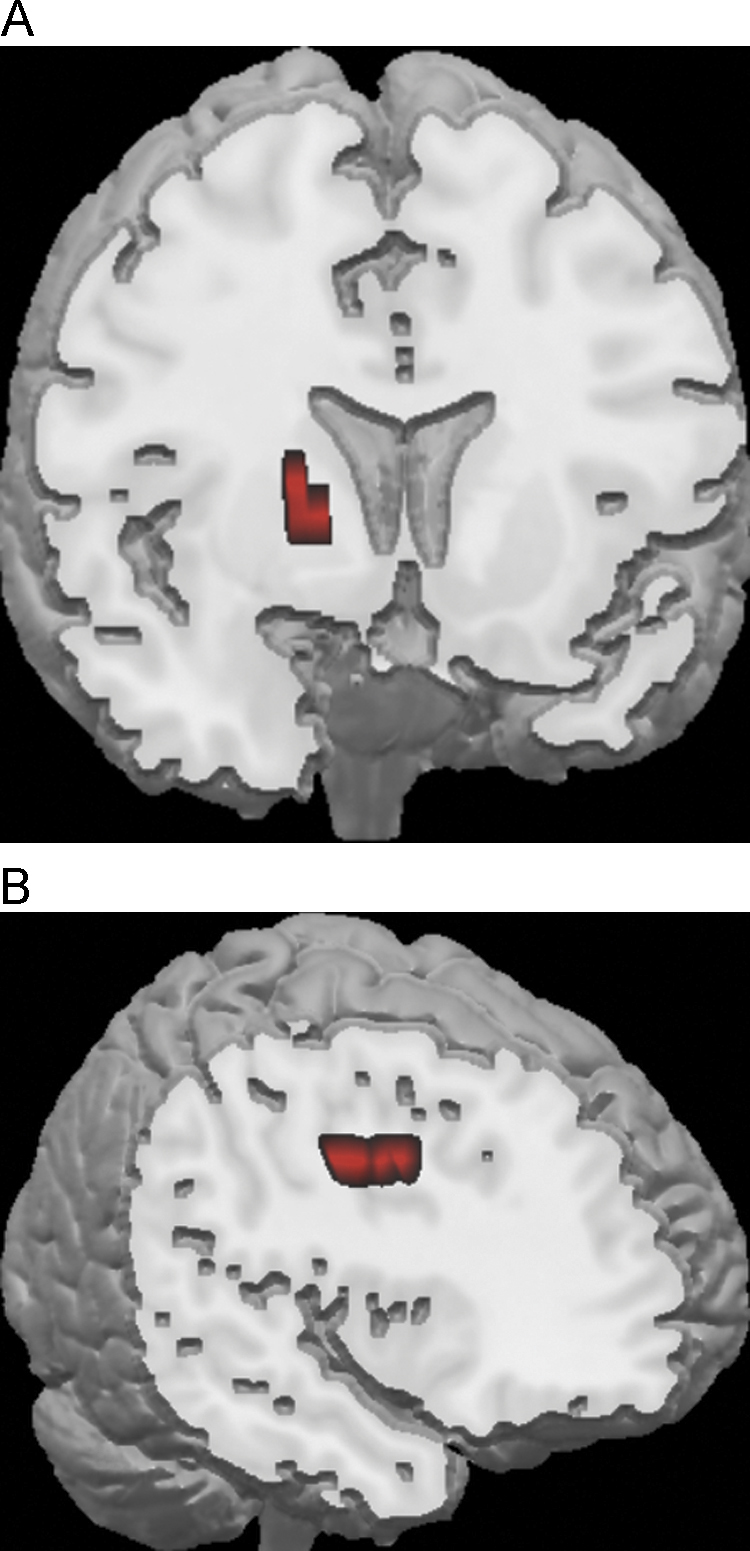
Visualization of the main differential regions for alexithymia severity. *Note:* — Neurological convention. Panel A, left globus pallidus externus, differential region for happy facial expression. Panel B, left dorsal anterior cingulate (BA 24), differential region for sad facial expression of emotion.

To further investigate the emotion-regulatory brain activation pattern exhibited by DPD, we examined, using hypothesis driven analyses, the relationships between neural and behavioral response to emotional stimuli in DPD and NC. The detailed results are presented in Supplemental Tables 1–4. To identify those regions, in which the NC and DPD groups significantly differ, we computed for each emotion and TAS subscale differential regression images (happy facial expressions, Table 2; sad emotional stimuli, Table 3); DPD and NC groups differed significantly in the following regions: For total TAS-20 score, right globus pallidus externus **(**Fig. 2, Panel A), and left dorsal ACC (BA 24) **(**Fig. 2**,** Panel B).

**Table 2:** Comparison of regression slopes depersonalization disorder>normal control subjects in happy expression intensities Alexithymia and composite taxons.

Region	Hemisphere	BA	Mass	*X*	*Y*	*Z*	*p*-Value
*TAS-*20 *Alexithymia level*							
Nucleus globus pallidus	L		492.0	−14	4	4	0.0255
*F1 Difficulty Identifying Feelings*							
Anterior insula	*R*		492.0	36	4	−7	0.0346
*F2 Difficulty describing feelings*							
Dorsal anterior cingulate	*L*	24	590.4	−10	18	20	0.0174
*F3 Externally oriented thinking*							
Paracingulate gyrus	*L*	32	590.4	−10	48	4	0.0104

*Note*: *Mass* volume in mm^3^, *BA* Brodmann area, *XYZ* Talairach coordinates, *p*-value tested against 50 random permutations.

**Table 3:** Comparison of regression slopes depersonalization disorder>normal control subjects in sad expression intensities Alexithymia and composite taxons.

Region	Hemisphere	BA	Mass	*X*	*Y*	*Z*	*p*-Value
*TAS-*20 *Alexithymia level*							
Dorsal anterior cingulate	*L*	24	590.4	−8	4	31	0.0087
*F*1 *Difficulty identifying feelings*							
Anterior insula	*L*		393.6	−39	11	9	0.0109
*F*2 *Difficulty describing feelings*							
Posterior cingulate	*L*	31	393.6	−4	59	31	0.0288
*F*3 *Externally oriented thinking*							
Orbital gyrus	*R*	10	492.0	−22	52	−2	0.0096

*Note*: *Mass* volume in mm^3^, *BA* Brodmann area, *XYZ* Talairach coordinates, *p*-value tested against 50 random permutations.

### 3.6 Groupwise signal levels and functional connectivity

**Fig. 3:**
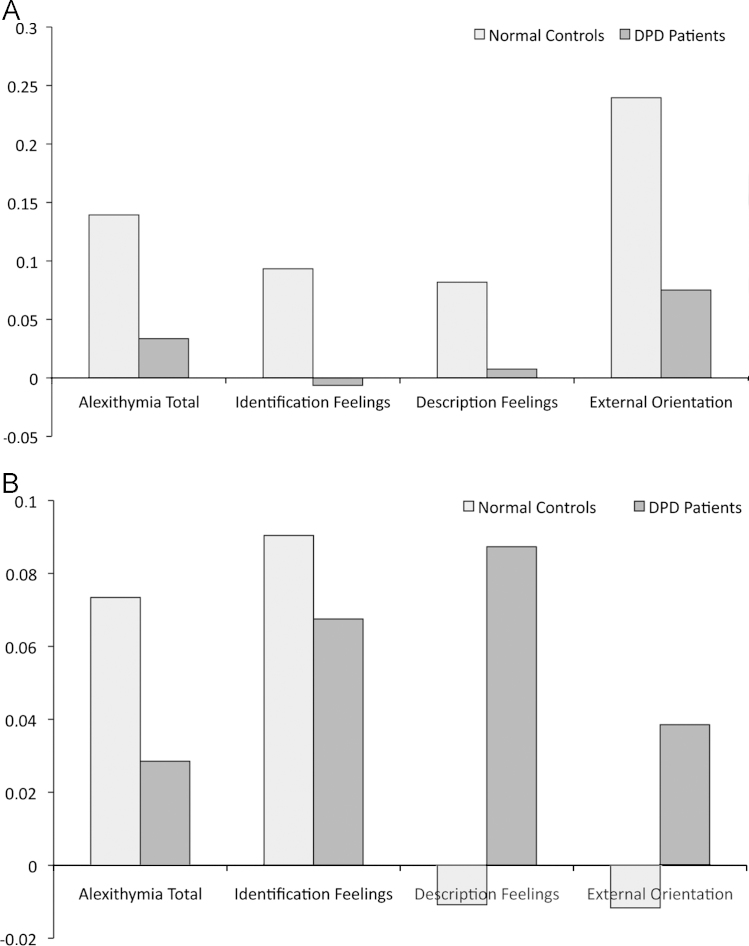
(A) BOLD signal levels from differential regions in happy condition. (B) BOLD signal levels from differential regions in sad condition.

The differential main clusters exhibiting the most significant group differences for the TAS-20 and its subscales were tested for functional connectivity (signal levels in Fig. 3A and B). We used the signal levels from these regions, in which the two groups most significantly differ in one alexithymia trait, to test for functional connectivity in terms of their inter-correlations (non-parametric exact point-probabilites were used to determine significances to compensate for small *N*): Happy NC: TAS-20 & F1 (*r*=0.987; *p*<0.0001), TAS-20 & F2 (*r*=0.953; *p*<0.0001), TAS-20 & F3 (*r*=0.981; *p*<0.0001), F1 & F2 (r=0.919; *p*<0.0001), F1 & F3 (*r*=0.851; *p*<0.0001), F2 & F3 (*r*=0.995; *p*<0.0001); sad NC: TAS-20 & F1 (*r*=0.608; *p*<0.001), TAS-20 & F2 (*r*=0.339; *p*<0.0001), TAS-20 & F3 (*r*=0.343; *p*<0.0001), F1 & F2 (*r*=0.533; *p*<0.0001), F1 & F3 (*r*=0.239; *ns*), F2 & F3 (*r*=0.347; *p*<0.0001); happy DPD: TAS-20 & F1 (*r*=0.163; *ns*), TAS-20 & F2 (*r*=0.245; *ns*), TAS-20 & F3 (*r*=0.267; *ns*), F1 & F2 (*r*=0.617; *p*<0.0001), F1 & F3 (*r*=0.404; *p*<0.0001), F2 & F3 (*r*=0.775; *p*<0.0001); sad DPD: TAS-20 & F1 (*r*=0.564; *p*<0.0001), TAS-20 & F2 (*r*=0.513; *p*<0.0001), TAS-20 & F3 (*r*=0.234; *ns*), F1 & F2 (*r*=0.269; *ns*), F1 & F3 (*r*=0.091; *ns*), F2 & F3 (*r*=0.662; *p*<0.0001). The indication\s for functional connectivity is that the numbers of significant paths for NC are 6 and 5; significant paths for DPD 3 and 3 (happy and sad, respectively). It can therefore be stated that, under emotional stimulation, DPD show reduced connectivity among regions discriminating them from healthy controls with regard to different alexithymia traits.

## 4 Discussion

The present study tested the assumption that the alexithymia trait contributes to the clinical diagnosis of DPD, and also shares cerebral mechanisms of emotion dysregulation with DPD. In the current study, we correlated neural responses during processing of three intensities of facial expressions of happy and sad emotion with self-reported alexithymia level, as well as with its three factor-analytic dimensions. In a novel statistical approach, we computed the regression slopes between clinical scales and BOLD responses and compared these between the DPD group and a matched healthy control group. We then identified brain regions with significant differences in regression slopes between the two groups. Functional connectivity was investigated for the differential regions in each of the two groups.

Behaviorally, we were able to support the hypothesis that alexithymia severity contributes substantially to the clinical diagnosis of DPD. The DPD group investigated in this study comprised mainly mid-to-high-alexithymic individuals. The analyses that we present reveal that alexithymia predicts DPD and uniquely explains 38% in the variance of diagnoses made by expert clinicians. Regarding abnormal processing of emotion in DPD, the regions indicating significant differences in alexithymia severity for happy emotion were the left globus pallidus externus, and the left dorsal anterior cingulate (BA 24) for sad emotion. The ascertained brain regions with altered responses in alexithymia are consistent with our hypotheses (i) to (vi), i.e. with the notion that they are associated with interoception, monitoring and reflection of internal states, and are in line with several previous studies. Overall, the pattern of changes that we observe is very similar to the “alexithymia regions” described for emotional memory tasks in PTSD patients, perhaps owing to earlier findings of a traumatogenic origin of DPD (Frewen et al., 2006, Frewen et al., 2008, Simeon et al., 2009). Moving to our prediction of co-involvement of affective regions of the pain neuromatrix (hypothesis v), we observed insular and ACC involvement, but not that of other regions of the pain matrix. For the three facets of alexithymia (hypothesis vi), as reflected by its subscales F1–F3, we can state that our findings of anterior insula, ACC, and orbital gyrus support the assumption of greatest group differences in interoceptive regions, and regions involved in emotion regulation. Regarding hypothesis (vii), we observed non-interoceptive regions in the DPD patients, supporting the assumption of alternate regions engaged in emotion processing—possibly explaining “emotional numbing”.

It is, however, important to note that the identified regions may not only reflect abnormal emotional introspection, but may also be related to reduced face-scanning abilities that have been shown to be associated with higher alexithymia severity (Bird et al., 2011). It is these peculiarities that may perhaps best explain why structures such as the globus pallidus emerged as central regions of differences in face processing. For happy and sad emotion intensities, the differentially responding regions for the subscale Identification of Feelings (TAS-20 F1) were anterior insula, left and right, respectively. The subscale Description of Feelings (TAS-20 F2) had significant slope differences in left anterior (BA 24) and left posterior (BA 31) cingulate gyrus, respectively. The regions showing group differences in BOLD/scale regression slopes for the subscale “Externally Oriented Concrete Thinking” (TAS-20 F3) were left paracingulate (BA 32) and right orbital gyrus (BA 10), respectively. We will briefly discuss these regions with regard to recent findings.

Globus pallidus activation is frequently seen in anger and disgust processing (Mataix-Cols et al., 2008), and lesions to it may cause DPD-like symptoms such as social withdrawal, anhedonia, an inability to feel, emotional blunting, emotional amnesia, and dementia-like inattention (Vijayaraghavan et al., 2008). Considering DPD, the globus pallidus has also been found to be involved in the cognitive generation of affect, and supporting trait anxiety intensity (Malhi et al., 2004). The ventral sector of the paracingulate gyrus is a region devoted to facial memory and affective other-person knowledge (Todorov et al., 2007). Previous studies have indicated its function in the assessment of social reward from personal interaction (Walter et al., 2005), and in other-related emotion reflection (Berthoz et al., 2002). PCC regions have long been associated with interoceptive processing and social cognition, more recently, as part of the self-related default-mode network, and for decision-making (Immordino-Yang et al., 2009, von dem Hagen et al., 2009, Andrews-Hanna et al., 2010). The anterior insula, which is also commonly found to be activated during visual emotion processing (Fusar-Poli et al., 2010), is part of the pain neuromatrix, devoted to homeostatic control, bodily risk assessment, and part of the defense system (Mobbs et al., 2009, Xue et al., 2010). The orbital gyrus (BA 10), a key region in emotional intensity regulation, is active during processing of happy and sad autobiographic memories (Markowitsch et al., 2003). The regions indicating different association levels between alexithymia scores and BOLD found in our experiments encompass several regions involved in interoception with an emphasis on emotional reflection, as one key characteristic of alexithymia (Berthoz et al., 2002).

The limitation of the present study lies in the relatively small sample size owing to the comparative rarity of DPD as a primary diagnosis. While we have no reason to suspect statistical bias due to co-morbidity, some confounding by possible medication effects in three patients cannot be excluded completely. Another limitation is that we had not administered an affect scale as an additional self-report control measure.

Our study represents the first investigation of alexithymia brain mechanisms in DPD. In terms of future work, it is of interest that the regions differentiating the DPD from NC reported here resemble regions described in activations found in PTSD sufferers. A fruitful approach would therefore be to carry out respective clinical group comparisons. Further possible research directions would consist of experimental investigations of deliberate emotion suppression and/or modification, internal sensations and/or pain processing of DPD patients, to further establish abnormal mechanisms in this condition.

## Role of the funding source

Funding for this study was provided by The Wellcome Trust, The Pilkington Trust, and by the British Council/German Academic Exchange Service. None of these funding bodies had a role in study design, in the collection, analysis and interpretation of data; in the writing of the report; and in the decision to submit the paper for publication.

## Contributors

E.L. and M.L.P conceived the study, E.L. coordinated the study, analyzed the data, and drafted the manuscript. All authors have read and approved. E.L., S.A.S. and M.L.P. devised the paradigm. M.S. examined the DPD patients. V.P.G. and M.J.B. assisted with data analyses, wrote custom software scripts, provided methodological advice, method descriptions, and helped editing the manuscript. S.C.R.W. supervised the imaging protocol and data collection. All authors contributed to, revised and approved the final manuscript.

## Footnotes

Appendix A Supplementary data associated with this article can be found in the online version at 10.1016/j.pscychresns.2013.05.006.
