# Supplementary material for: Interoceptive–reflective regions differentiate alexithymia traits in depersonalization disorder
Source: Psychiatry Res. 2013 Oct 30;214(1):66–72. doi: 10.1016/j.pscychresns.2013.05.006 (PMC4024664; doi:10.1016/j.pscychresns.2013.05.006)
Supplement: Supplementary file 1 — Supplementary data [file mmc1.docx]

**Supplementary Online Information**

**Interoceptive-reflective regions differentiate alexithymia traits in depersonalization disorder**

Erwin Lemche ^a^***, Michael J. Brammer ^b^, Anthony S. David ^a^, Simon A. Surguladze ^a^, Mary L. Phillips ^c^, Mauricio Sierra ^a^, Steven C.R. Williams ^b^, and Vincent P. Giampietro ^b^

^a^ *Section of Cognitive Neuropsychiatry, Institute of Psychiatry, De Crespigny Park, London SE5 8AF, UK*

^b^ *Department of Neuroimaging, Institute of Psychiatry, De Crespigny Park, London SE5 8AF, UK*

^c^ *Western Psychiatric Institute and Clinic, University of Pittsburgh, 3811 O’Hara Street, Pittsburgh, PA 15213, USA*

"This material supplements but does not replace the content of the peer-reviewed paper published in *Psychiatry Research: Neuroimaging*"

## **Implicit Facial Expression Neuroimaging Tasks**

The display of facial stimuli in the series Pictures of Facial Affect (POFA) (Ekman and Friesen, 1976) is based on specified facial muscle movements according to the Facial Action Coding System (Ekman and Friesen, 1978a). Facial identities (6 females, 4 males) are depicted that pose facial muscle configurations which have been demonstrated to constitute inter-culturally universal expressions (Ekman et al., 1982) for basic emotions (Ekman, 1992a; Ekman, 1992b). The original POFA stimuli have been computer-morphed to conform artificially to 6 levels of expression intensity (0 through 150%). The different identities of the computer-enhanced POFA stimuli (FEEST) (Young et al., 2002) are only discernible by means of features indicating sex, emotion category, and expression intensity. Previous studies have demonstrated that neural responses to emotional stimuli are dependent on the nature of the task performed during viewing of the stimuli (Lange et al., 2003). Usually, implicit tasks (e.g. sex decision) have sparser overall responses and are thus more conservative. Subjects in this study were requested to decide upon the sex of each face and press one of two buttons accordingly with their index and middle fingers. fMRI data were acquired for each emotional expression at three different intensities (0%-50%-100%) in a randomized order. In each implicit emotion processing task, 72 stimuli in total were presented with an inter-stimulus interval (ISI) of 2,000 ms. The order of the experiments was counterbalanced across all subjects, and both were embedded into sessions with other experiments. Subjects responded by button press and reaction times and response type were recorded. The computer presentation of the experiments was performed through back-projection using an LCD-projector and a translucent screen, which subjects watched in supine position through a mirror mounted on the MRI head coil. The display subtended a visual angle of ≥8°.

***fMRI Data Processing***

The statistical inference software package Brain Activation Mapping (*XBAM*, Version 4.1; Brain Image Analysis Unit, Institute of Psychiatry, London, UK; *www.brainmap.it*) was used to analyze the EPI images. The data were first realigned (Bullmore et al., 1999a) to minimize motion related artifacts and smoothed using a Gaussian filter (FWHM 7.2 mm). Responses to the experimental paradigm were then detected by time-series analysis using Gamma variate functions (peak responses at 4 and 8 s) to model the BOLD response. The analysis was implemented as follows: First, each experimental condition was convolved separately with the 4 and 8 s Poisson functions to yield two models of the expected hemodynamic response to that condition. The weighted sum of these two convolutions that gave the best fit to the time series at each voxel was then computed. This weighted sum effectively allows voxel-wise variability in time to peak hemodynamic response. In order to constrain the possible range of fits physiologically plausible BOLD responses, the constrained fitting procedure suggested by Friman (Friman et al., 2003) was adopted. Following this fitting operation, a goodness of fit statistic was computed at each voxel. This was the ratio of the sum of squares (SSQ) of deviations from the mean intensity value due to the model (fitted time series) divided by the sum of squares due to the residuals (original time series minus model time series). The percentage BOLD signal change at each voxel was also calculated. This was ((*fitmax* – *fitmin*)/mean signal intensity) * 100, where *fitmax* and *fitmin* were the maximum and minimum values of the fitted response for the time series in question. In order to sample the distribution of SSQ ratio under the null hypothesis that observed values of SSQ ratio were not determined by experimental design (with minimal assumptions), the time series at each voxel was permuted using the Daubechies wavelet resampling method described in detail in Bullmore (Bullmore et al., 2001) and extended by Breakspear (Breakspear et al., 2003; Breakspear et al., 2004). Combining the randomized data over all voxels yields the distribution of SSQ ratio under the null hypothesis. A test that any given voxel is activated at any required type I error can then be carried out by obtaining the appropriate critical value of SSQ ratio from the null distribution. For example, SSQ ratio values in the observed data lying above the 99^th^ percentile of the null distribution have a probability under the null hypothesis of *p*<0.01. We have shown that this permutation method gives very good type I error control with minimal distributional assumptions (see Bullmore et al., 2001; Breakspear et al., 2003).

# *Group Mapping and Between-Group Contrast Differences*

The median observed SSQ ratio over all subjects at each voxel (median values were used to minimize outlier effects) can then be tested at each intracerebral voxel in standard space (Talairach and Tournoux, 1988) against a critical value of the permutation distribution for median SSQ ratio ascertained from the spatially transformed wavelet-permuted data (Brammer et al., 1997). Image-wise expectation of the number of false positive clusters under the null hypothesis is set for each analysis at <0.5. Analysis of variance was carried out on the SSQ ratio maps in standard space by first computing the difference in median SSQ ratio between groups at each voxel. Subsequent inference of the probability of this difference under the null hypothesis was made by reference to the null distribution obtained by repeated random permutation of group membership and re-computation of the difference in median SSQ ratios between the two groups obtained from the resampling process. Cluster-level maps were then obtained as described above. Supplemental Text 901 words

# Supplementary Table 1. Correlations with Behavioral Measures for Depersonalization Disorder in Happy Condition

Alexithymia and composite subscales

Region Hemisphere BA Mass *X* *Y* *Z* *p*-Value

*Alexithymia level*

Hypothalamus R 393.6 2 -2 -8 0.0000

*F1 Difficulty Identifying Feelings*

Cerebellum L 1406.0 -4 -60 26 0.0000

*F2 Difficulty Describing Feelings*

Hippocampus L 590.4 -35 -17 -13 0.0001

*F3 Externally-Oriented Thinking*

Mesial Superior Frontal Gyrus R 10 1771.2 11 62 4 0.0000

Note: — *Mass* volume in mm^3^, *BA* Brodmann Area, *XYZ* Talairach Coordinates, *p*-Value tested against 50 random permutations.

# Supplementary Table 2. Correlations Behavioral Measures for Depersonalization Disorder in Sad Condition

Alexithymia and composite subscales

Region Hemisphere BA Mass *X* *Y* *Z* *p*-Value

*Alexithymia level*

Retrosplenial Cortex R 35 492.0 18 -37 -7 0.0000

*F1 Difficulty Identifying Feelings*

Anterior Temporal Pole R 38 787.2 -36 11 -18 0.0000

*F2 Difficulty Describing Feelings*

Paracingulate Gyrus L 32 1180.8 7 48 9 0.0039

*F3 Externally-Oriented Thinking*

Mesial Superior Frontal Gyrus R 10 2066.4 10 48 20 0.0000

Note: — *Mass* volume in mm^3^, *BA* Brodmann Area, *XYZ* Talairach Coordinates, *p*-Value tested against 50 random permutations.

# Supplementary Table 3. Correlations in Normal Control Subjects in Happy Expression with Behavioral Measures

Alexithymia and composite subscales

Region Hemisphere BA Mass *X* *Y* *Z* *p*-Value

*Alexithymia level*

Cerebellum L 1771.2 -14 -37 -40 0.0000

*F1 Difficulty Identifying Feelings*

Cerebellum L 2066.4 -14 -74 -40 0.0000

*F2 Difficulty Describing Feelings*

Cerebellum L 1672.8 -14 -48 -45 0.0000

*F3 Externally-Oriented Thinking*

Cerebellum R 1869.6 18 -74 -29 0.0000

Note: — *Mass* volume in mm^3^, *BA* Brodmann Area, *XYZ* Talairach Coordinates, *p*-Value tested against 50 random permutations.

# Supplementary Table 4. Correlations with Behavioral Measures for Normal Control Subjects in Sad Condition

Alexithymia and composite subscales

Region Hemisphere BA Mass *X* *Y* *Z*  *p*-Value

*Alexithymia level*

Inferior Frontal Gyrus R 47 1377.6 32 29 -18 0.0000

*F1 Difficulty Identifying Feelings*

Transverse Temporal Gyrus R 42 2164.8 32 -29 14 0.0000

*F2 Difficulty Describing Feelings*

Temporal Operculum R 22 1771.2 54 -11 9 0.0000

*F3 Externally-Oriented Thinking*

Superior Temporal Gyrus L 21 1547.4 -50 -36 9 0.0000

Note: — *Mass* volume in mm^3^, *BA* Brodmann Area, *XYZ* Talairach Coordinates, *p*-Value tested against 50 random permutations.

371 words

**Supplementary References**

Brammer, M.J., Bullmore, E.T., Simmons, A., Williams, S.C.R., Grasby, P.M., Howard, R.J., Woodruff, P.W.R., Rabe-Hesketh, S., 1997. Generic brain activation mapping in functional magnetic resonance imaging: A nonparametric approach. Magnetic Resonance Imaging 15, 763-770.

Breakspear, M., Brammer, M.J., Bullmore, E.T., Das, P., Williams, L.M., 2004. Spatiotemporal wavelet resampling for functional neuroimaging data. Human Brain Mapping 23, 1-25.

Breakspear, M., Brammer, M.J., Robinson, P.A., 2003. Construction of multivariate surrogate sets from nonlinear data using the wavelet transform. Physica D: Nonlinear Phenomena 182, 1-22.

Bullmore, E.T., Brammer, M.J., Rabe-Hesketh, S., Curtis, V.A., Morris, R.G., Williams, S.C.R., Sharma, T., McGuire, P.K., 1999a. Methods for diagnosis and treatment of stimulus-correlated motion in generic brain activation studies using fMRI. Human Brain Mapping 7, 38-48.

Bullmore, E.T., Long, C., Suckling, J., Fadili, M.J., Calvert, G., Zelaya, F., Carpenter, T.A., Brammer, M.J., 2001. Color noise and computational inference in neurophysiological (fMRI) time series analysis: Resampling methods in time and wavelet domains. Human Brain Mapping 12, 61-78.

Ekman, P., 1992a. Are there basic emotions? Psychological Review 99, 550-553.

Ekman, P., 1992b. An argument for basic emotions. Cognition and Emotion 6, 169-200.

Ekman, P., Friesen, W., 1978a. The facial action coding system. Consulting Psychologists Press, Palo Alto, CA.

Ekman, P., Friesen, W.V., 1976. Pictures of facial affect. Consulting Psychologists Press, Palo Alto, CA.

Ekman, P., Friesen, W.V., Ellsworth, P., 1982. What are the similarities and differences in facial behavior across cultures?, in: Ekman, P. (Ed.), Emotions in the Human Face, 2 ed. Cambridge University Press, Cambridge, UK.

Friman, O., Borga, M., Lundberg, P., Knutson, H., 2003. Adaptive analysis of fMRI data. NeuroImage 19, 837-845.

Lange, K., Williams, L.M., Young, A.W., Bullmore, E.T., Brammer, M.J., Williams, S.C.R., Gray, J.A., Phillips, M.L., 2003. Task instructions modulate neural responses to fearful facial expressions. Biological Psychiatry 53, 226-232.

Talairach, J., Tournoux, P., 1988. Co-planar stereotaxic atlas of the human brain. Thieme Medical Publishers, Stuttgart, Germany.

Young, A.W., Perrett, D.I., Calder, A.J., Ekman, P., 2002. Facial expressions of emotion: stimuli and test (FEEST). Thames Valley Test Company, Bury St. Edmunds, UK.

Supplementary References 337 words
